# Treatment of Infantile Inflammatory Bowel Disease and Autoimmunity by Allogeneic Stem Cell Transplantation in LPS-Responsive Beige-Like Anchor Deficiency

**DOI:** 10.3389/fimmu.2017.00052

**Published:** 2017-01-31

**Authors:** Shahrzad Bakhtiar, Laura Gámez-Díaz, Andrea Jarisch, Jan Soerensen, Bodo Grimbacher, Bernd Belohradsky, Klaus-Michael Keller, Christoph Rietschel, Thomas Klingebiel, Sibylle Koletzko, Michael H. Albert, Peter Bader

**Affiliations:** ^1^Division for Pediatric Stem-Cell Transplantation and Immunology, University Hospital Frankfurt, Frankfurt/Main, Germany; ^2^Center for Chronic Immunodeficiency, University Hospital, Freiburg, Germany; ^3^Dr. v. Hauner Children’s Hospital, Ludwig-Maximilians-University, Munich, Germany; ^4^German Clinic for Diagnostics (DKD) Helios Klinik, Wiesbaden, Germany; ^5^Clementine Children’s Hospital, Frankfurt/Main, Germany

**Keywords:** infantile IBD, autoimmune cytopenia, lipopolysaccharide-responsive beige-like anchor gene, stem cell transplantation, vitiligo

## Abstract

Inflammatory bowel disease (IBD) in young children can be a clinical manifestation of various primary immunodeficiency syndromes. Poor clinical outcome is associated with poor quality of life and high morbidity from the complications of prolonged immunosuppressive treatment and malabsorption. In 2012, mutations in the lipopolysaccharide-responsive beige-like anchor (LRBA) gene were identified as the cause of an autoimmunity and immunodeficiency syndrome. Since then, several LRBA-deficient patients have been reported with a broad spectrum of clinical manifestations without reliable predictive prognostic markers. Allogeneic hematopoietic stem cell transplantation (alloHSCT) has been performed in a few severely affected patients with complete or partial response. Herein, we present a detailed course of the disease and the transplantation procedure used in a LRBA-deficient patient suffering primarily from infantile IBD with immune enteropathy since the age of 6 weeks, and progressive autoimmunity with major complications following long-term immunosuppressive treatment. At 12 years of age, alloHSCT using bone marrow of a fully matched sibling donor—a healthy heterozygous LRBA mutant carrier—was performed after conditioning with a reduced-intensity regimen. During the 6-year follow-up, we observed a complete remission of enteropathy, autoimmunity, and skin vitiligo, with complete donor chimerism. The genetic diagnosis of LRBA deficiency was made post-alloHSCT by detection of two compound heterozygous mutations, using targeted sequencing of DNA samples extracted from peripheral blood before the transplantation.

## Introduction

Enteropathy is one of the major clinical symptoms of several primary immunodeficiencies (PIDs) (1), such as immunodysregulation, polyendocrinopathy, enteropathy, X-linked (IPEX) syndrome, and chronic granulomatous disease (CGD), as well as the IL-10/IL-10-receptor (2), CD25 (3), and Stat5b deficiency (4). Recently, several novel PIDs, resulting primarily in autoimmunity and immune dysregulation, were added to this group. Examples are the disorders caused by the deficiency of the lipopolysaccharide-responsive beige-like anchor (LRBA) (5, 6), the cytotoxic T-lymphocyte-associated protein 4 (CTLA-4) (7), and the mucosa associated lymphoid tissue lymphoma translocation 1 (8), as well as the syndromes caused by gain-of-function mutations of the signal transducer and activator of transcription 1 (STAT1) (9) and STAT3 (10, 11).

Among these disorders, LRBA and CTLA-4 deficiencies seem to have similar symptoms; patients of both develop primarily heterogeneous multiorgan autoimmunity. Inflammatory bowel disease (IBD) occurs in about 70% of affected patients. Other symptoms include hypogammaglobulinemia, autoimmune cytopenia, endocrine manifestations, and lymphoproliferation (7, 12, 13).

Lipopolysaccharide-responsive beige-like anchor is involved in critical cellular processes, such as apoptosis and autophagy, and plays a role in immune system regulation (14). There is evidence that LRBA influences CTLA-4 homeostasis and, by extension, the suppressive capacity of regulatory T-cells (Tregs) by preventing CTLA-4 degradation in endocytic lysosomes (15). Some authors classify LRBA and CTLA-4 deficiencies as “Treg-opathies” with an IPEX-like disease phenotype (16). Notably, and in contrast to patients with IPEX or LRBA deficiency, CTLA-4-deficient patients seem to show a later disease manifestation during their second decade of life. However, the clinical manifestations and the severity of the symptoms among affected patients remain heterogeneous (12, 13, 17–19). Moreover, these disorders lack a clear genotype–phenotype correlation, and corresponding prognostic and predictive biomarkers have not yet been established.

Severely affected patients do not respond to standard immunosuppressive treatment, such as methotrexate, sirolimus, azathioprine, cyclosporine A, and steroids (12, 13). For patients with IBD as their major symptom, malabsorption and failure to thrive are common complications, compromising the outcome of the disease and the patient’s quality of life. Frequently, multiple immunosuppressive treatments result in infectious and non-infectious complications, exacerbating the disease.

For some well-defined PIDs, such as the severe combined immunodeficiency (SCID) and CGD, there are clear indications for early allogeneic hematopoietic stem cell transplantation (alloHSCT) or, in some cases, gene therapy. In contrast, there are often no clear indications for early alloHSCT in patients with novel immune system-dysregulative PIDs, as these exhibit heterogeneous disease manifestations and a lack of genotype–phenotype correlation.

To date, three reports are available on alloHSCT-treated severely affected patients from consanguineous families harboring homozygous LRBA mutations (20–22). Herein, we describe the detailed clinical course of the disease in a Caucasian child diagnosed with a therapy-refractory infantile IBD with autoimmunity, caused by two compound heterozygous LRBA mutations. The patient underwent successful alloHSCT at 12 years of age with non-myeloablative conditioning. During the 6-year follow-up, we observed a stable engraftment and complete donor chimerism. Major symptoms of LRBA deficiency, enteropathy, and autoimmune cytopenia resolved completely, while partial resolution of vitiligo was evident.

## Methods

### Case Report

**Figure 1:**
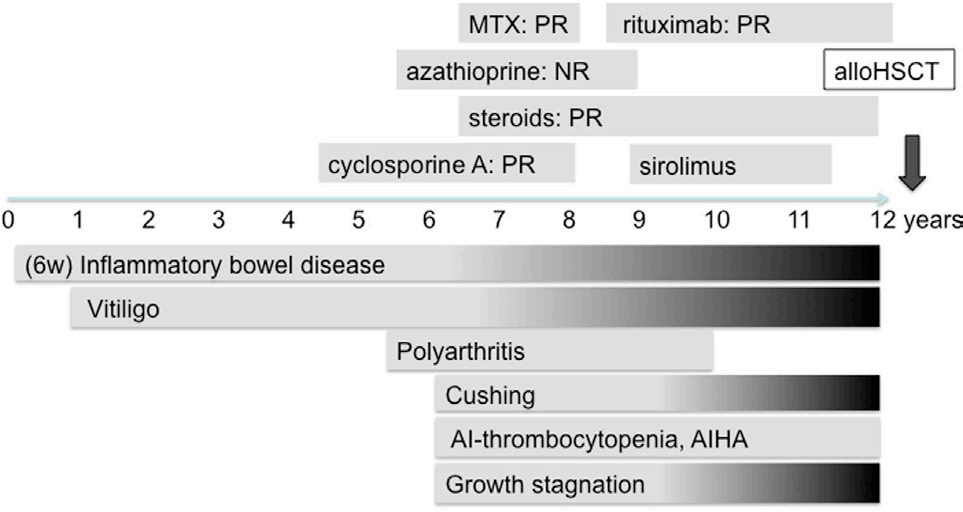
**Clinical course of the disease and immunosuppressive treatments provided to the lipopolysaccharide-responsive beige-like anchor-deficient patient**. PR, partial remission; NR, non-response. Gray shading indicates progressive symptoms.

**Figure 2:**
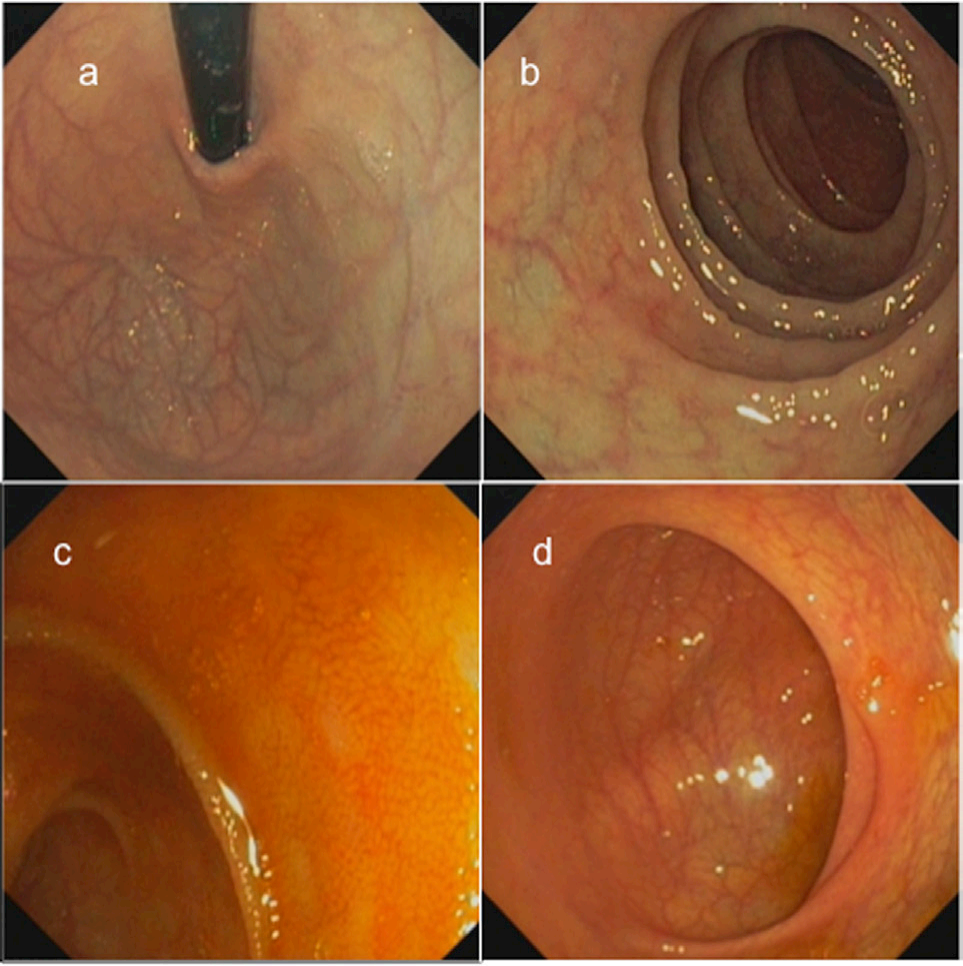
**Gastroscopic findings of the patient showing atrophic gastritis with visible vessels in the corpus and fundus ventriculi (A), shiny mucosa with few villi but without macroscopic lesions in the duodenum (B), terminal ileum without visible lymph follicles (C), and normal cecum with ileocecal valve (D)**.

**Figure 3:**
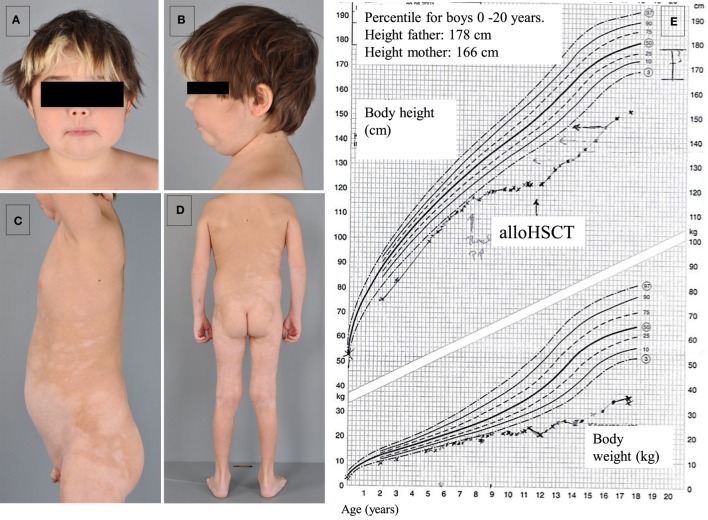
**Development of secondary Cushing’s syndrome (A,B), vitiligo (C,D), and complete growth stagnation with post-alloHSCT growth acceleration (E)**.

The patient, currently 18 years old, first presented chronic diarrhea and failure to thrive at the age of 6 weeks. Gastroscopy revealed changes suggesting celiac disease. He was started on a gluten-free diet, without amelioration of the symptoms. Progressive vitiligo appeared at the age of 1 year. At 4.5 years of age, immunosuppressive treatment with cyclosporine A was started, resulting in the partial remission of enteropathy (Figure 1). The patient’s IBD was progressive. At 12 years of age, gastroscopy including histological assessment of gastric biopsies revealed an antiparietal cell-positive autoimmune gastritis characterized by atrophy, metaplasia, and endocrine cell hyperplasia (Figures 2A,B), providing an explanation for patient’s vitamin B12 deficiency refractory to oral substitution. Colonoscopy showed autoimmune colitis with graft versus host-like features with lymphofollicular hyperplasia, increased epithelial regeneration, and increased apoptosis without any signs of infectious agents (Figures 2C,D). The patient developed aseptic polyarthritis affecting both knees and ankles at 5 years, and autoimmune thrombocytopenia at 7 years. At this stage, the immunosuppressive treatment included high doses of steroids, sirolimus, azathioprine, and multiple courses of rituximab; this regimen resulted in a short-term partial remission of the symptoms. By the age of 12 years, three episodes of autoimmune hemolytic anemia had occurred. A combination of rituximab, sirolimus, and an escalated dosage of steroid (prednisolone, 2 mg/kg/day) was required to keep the symptoms under control. The patient developed severe secondary Cushing’s syndrome and exhibited complete stagnation of growth (Figure 3). By the age of 12, a body weight of 23 kg (4 kg below P3), a height of 122 cm (14 cm below P3), and a body mass index of 16 were documented (Figures 3A–E). Assessment of bone age according to Greulich and Pyle revealed a significant developmental delay (5 years) and severe osteopenia.

The patient’s immunological workup at 8 years of age (on prednisolone and azathioprine) showed normal levels of immunoglobulins (Igs) G, M, A, and E; low levels of tetanus vaccine antibodies, and; moderate T-cell lymphocytopenia (CD3^+^: 1,230 cells/μL [non-response (NR): 1,100–5,900/μl]) with normal distribution of CD4^+^ and CD8^+^ T-cells, naive CD4^+^ (CD4^+^CD45RA^+^CD62L^+^) T-cells, and double-negative T-cells (CD3^+^CD4^−^CD8^−^TCRα/β and γ/δ). Tregs (CD4^+^CD25^+^Foxp3^+^) were reduced [2.5% (NR > 5%)]. T-cell proliferation, assessed by phytohemagglutinin (PHA) stimulation of CD4^+^ and CD8+ T-cells, and the V-beta repertoire of CD4^+^ and CD8^+^ T-cells were within normal ranges. Also the counts of NK cells (CD3^−^CD56^+^) and B-cells (CD19^+^), as well as those of the memory and class-switched memory B-cells (CD19^+^CD27^+^IgD^−^) were normal. Direct and indirect Coombs’ tests were positive with detectable anti-erythrocyte antibodies. Endocrine parameters, including thyroid and growth hormones, were unremarkable. Antinuclear antibodies as well as antibodies against lactoferrin, carboanhydrase I, carboanhydrase II, and parietal cells, were not detectable. High levels of the autoimmune enteropathy-related antigen [AIE-75, 187.4 (NR ratio < 10)] antibodies were detected in plasma using radioimmune precipitation assay, indicating an autoimmune enteropathy (Table 1).

**Table 1:** **Laboratory workup of the patient with lipopolysaccharide-responsive beige-like anchor deficiency**.

Lab results	Normal range	Patient
WBC	4–11/nl	8.2
Hb	11–15 g/l	7.3*
Thrombocytes	200–400/nl	**40^#^**
T-lymphocytes (CD3^+^)	700–4,200/nl	794*
Granulocytes	1.8–7.1/nl	2.1**
CD4^+^ T-cells	300–2,000/μl	425*
CD8^+^ T-cells	300–1,800/μl	313*
Naive CD4^+^ (CD4^+^CD45RA^+^CD62L^+^)	220–873/μl	254*
DNT (CD3^+^TCRg/d^+^CD4^−^CD8^−^)	<5%	1.7%*
Regulatory T-cells (CD4^+^CD25^+^FoxP3^+^)	5–10%	2.8%*
B-cells (CD19^+^)	200–1,500/μl	306*
PHA		Normal*
V-beta CD4^+^ and CD8^+^		Normal*
Switched memory B (CD19^+^CD27^+^IgD^−^)	>5%	8%*
Non-switched memory B (CD19^+^CD27^+^IgD^+^)	>5%	4%*
NK cells (CD3^−^CD56^+^)	60–900/μl	69
IgG	590–1,400 mg/dl	780**
IgM	50–317 mg/dl	60*
IgA	70–250 mg/dl	109**
IgE	<100 U/ml	1
Coombs test	−	++**
Anti-thrombocyte Ab	−	++**
Anti-granulocyte Ab	−	++**
TSH	0.5–3.6 mU/l	2.0*
fT4	0.9–1.6 ng/dl	1.2*
IgfBP3	2.3–8.3 µg/ml	3.4**
IGF-1	101–538 ng/ml	151**
Anti-TG Ab	<40 IU/ml	<40*
Anti-TPO Ab	<35 IU/ml	<35*
Anti-gliadin Ab	<15 U/ml	0.8*
Anti-lactoferrin Ab	<10	2.1*
Anti-carboanhydrase I Ab	−	−*
Anti-carboanhydrase II Ab	−	−*
ANA	−	−*
Antiparietal cell Ab	−	+**
AIE-75	<10	187.4*

*, **, and ^#^ indicate values obtained at 8, 9, and 11 years of age, respectively (no single age at which all tests had been done was available). − indicates non-detectable; +, ++, and +++ indicate weak, medium, and strong signals, respectively. PHA, phytohemagglutinin; DNT, double-negative T-cells; V-beta, T-cell receptor V-beta repertoire; NK, natural killer cells; TSH, thyroid-stimulating hormone; fT4, free thyroxine; IgfBP3, insulin-like growth factor-binding protein 3; IGF-1, insulin-like growth factor-1; TG, thyroglobulin; TPO, thyroperoxidase; ANA, antinuclear antibodies; AIE-75, autoimmune enteropathy antibody-75. Bold font is used for patient results outside the normal range.

### Genetic Workup

Both written parental consent and approval by the Ethics Committee of the University Hospital Frankfurt were obtained. Sanger sequencing of genomic DNA extracted from peripheral blood was performed to identify SCID-causing mutations in the RAG1, RAG2, DCLRE1C, LIG4, and XLF genes. Two heterozygous variants with unknown pathogenicity were detected in RAG1 and RAG2 (one variant for each gene). No disease-causing mutations were found in FOXP3. The clinical manifestation of the disease was evaluated as IPEX-like.

### LRBA Protein Detection by Flow Cytometry

Isolated peripheral blood mononuclear cells (PBMCs) were stimulated with 10 ng/µl of PHA (cat. L1668, Sigma-Aldrich, St. Louis, MO, USA) for 72 h. Intra cellular expression of LRBA in unstimulated and stimulated PBMCs was determined by flow cytometry. Briefly, 3 × 10^5^ cells were permeabilized and fixed using the BD Cytofix/Cytoperm solution (cat. 554715; BD Biosciences, Great Lakes, NJ, USA), stained with rabbit polyclonal anti-LRBA antibody (cat. HPA023597; Sigma-Aldrich), followed by PE-conjugated F(ab’)2 Donkey anti-Rabbit IgG (cat. 558416; BD Biosciences). Cells were analyzed on a FACSCanto II system (BD Biosciences). Data analysis was performed using FlowJo v10 (TreeStar Inc., Ashland, OR, USA). Mean fluorescence intensities were calculated and compared with those of non-related healthy controls.

### *LRBA* Mutation Detection

The identification of the genetic defect was performed on genomic DNA extracted from frozen PBMC samples collected 4 weeks prior to alloHSCT. Briefly, 225 ng of gDNA was digested and hybridized with a HaploPlex biotinylated probe library (Agilent Technologies, Santa Clara, CA, USA) in the presence of an indexing primer cassette for enrichment. After capturing and ligating the circularized target DNA-probe hybrids on streptavidin beads, targeted fragments were amplified by PCR. Sample barcodes were introduced during amplification for precise tracking. Elution, PCR amplification, and pooling of the targeted samples with different indexes were performed to prepare for multiplex sequencing on the Illumina MiSeq platform (Illumina, San Diego, CA, USA). On average, 99% of exon bases at a depth of at least 38 × were covered. The sequences were aligned to the human genome using the Agilent SureCall software (Agilent Technologies). LRBA mutations detected by next-generation sequencing were validated by Sanger sequencing. The PCR products were sequenced in both directions and analyzed with Sequencer v4.10.1 (Gene Codes Corporation, Ann Arbor, MI, USA).

## Results

### Transplantation Procedure

Severe enteropathy refractory to immunosuppressive therapy that necessitated parenteral nutrition, as well as the development of autoimmunity by the age of 12 years, justified alloHSCT as an experimental curative option, although genetically confirmed diagnosis was not available at that stage. Secondary symptoms following long-term steroid treatment, such as significantly reduced numbers of T-, B-, and NK cells, growth stagnation, and progressive Cushing’s syndrome, also constituted major indications for alloHSCT. A clinically healthy 15-year-old sibling sharing the patient’s human leukocyte antigen (HLA) served as the donor. The patient received unmanipulated bone marrow containing 12.9 × 10^6^ CD34^+^cells/kg and 36.2 × 10^6^ CD3^+^ cells/kg following a non-myeloablative conditioning regimen including fludarabine (5 × 40 mg/m^2^), melphalan (2 × 70 mg/m^2^), and thiotepa (2 × 5 mg/kg). Anti-thymocyte globulin (3 × 20 mg/kg) was used as serotherapy from day −4 to day −1. Graft versus host disease (GVHD) prophylaxis consisted of cyclosporine A (from day −1; dose was adjusted to achieve serum trough levels of 100–150 ng/ml) and low doses of methotrexate (10 mg/m^2^) on days +1 and +3 post-HSCT (Table 2). The conditioning regimen was tolerated well without any signs of organ toxicity. Two episodes of febrile neutropenia were observed on days −3 and +10, which were resolved with appropriate anti-infective treatments. No virus reactivation or opportunistic infections occurred.

**Table 2:** **Transplant characteristics**.

Transplant characteristics	
Donor	10/10 MSD
CMV status	Positive donor/negative recipient
Blood group	O Rh^+^ (both donor and recipient)
Stem cell source	Bone marrow
Cell dose/kg	
CD34^+^	12.9 × 10^6^
CD3^+^	36.2 × 10^6^
Conditioning regimen	Non-myeloablative
Fludarabin	5 × 40 mg/m^2^
Thiotepa	2 × 5 mg/kg
Melphalan	2 × 70 mg/m^2^
Serotherapy	
ATG	3 × 20 mg/kg
Time to engraftment (day)	
Neutrophils (ANC > 0.5 × 10^9^/nl)	17
Platelets (>20 × 10^9^/nl)	11
Thrombocytes (> 50/nl)	45
GCSF	5 µg/kg (days 14–20)
Transfusions post-transplant	
Red cells (U)	6
Platelets (U)	18
Graft versus host disease (GVHD) prophylaxis	Cyclosporine A (trough level 100–150 ng/ml)
GVHD	
Acute, grade	Skin I–II (limited)
Chronic	No
Chimerism (whole blood)	100% donor (day +19 onward)
Lansky performance score, last follow-up	100%
Follow-up (months)	72

### Engraftment, GVHD, and Chimerism

An uncomplicated engraftment was observed. Leukocyte and granulocyte recovery occurred on days +17 and +18, respectively. Thrombocyte recovery was observed on day +45 post-HSCT. The patient developed intermittent acute GVHD grade I–II, which was limited to the skin. No additional treatment was required. During the 6-year follow-up, no signs of *de novo* onset of acute or chronic GVHD were observed. The patient has remained off immunosuppression since day +70. Stable chimerism was observed in T-cells (100% of donor) and whole blood (100% of donor) beginning from day +19 onward.

### Clinical and Immunological Recovery

With respect to the clinical symptoms associated with the underlying disease, enteropathy and multisystemic autoimmunity resolved completely after alloHSCT. No further episodes of chronic diarrhea and malabsorption were observed. Autoimmune hemolytic anemia or cytopenia were not observed, either. During the 6-year follow-up, a partial re-pigmentation of the vitiligo was observed. The cushingoid habitus regressed and the patient gained height (Figure 3E). The patient was expected to achieve the constitutionally determined height. Following alloHSCT, the patient developed age-normal T-, B-, and NK cell counts, as well as Ig serum levels. Protective antibody titers to tetanus and hepatitis-B vaccines were observed.

### Post-Transplantation Establishment of the Underlying Diagnosis

**Figure 4:**
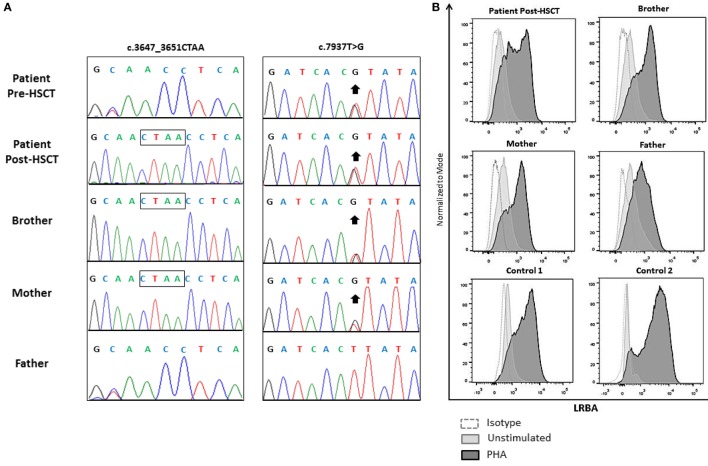
**Sequencing chromatograms of lipopolysaccharide-responsive beige-like anchor (LRBA) variants from genomic DNA extracted from the peripheral blood of the patient 4 weeks before and 1 year after HSCT (frozen DNA samples) as well as from genomic DNA isolated from the patient’s parents and brother**. Deleted nucleotides are denoted by a box. The missense mutation is indicated by an arrow **(A)**. LRBA expression levels in the patient post-alloHSCT, his clinically healthy parents and brother, and two non-related healthy controls. LRBA expression was measured in total peripheral blood mononuclear cells before and 3 days after phytohemagglutinin stimulation **(B)**.

We performed targeted sequencing for a screening panel of “common variable immune deficiency (CVID) candidate genes” (Table 3). The patient harbored two compound heterozygous mutations of the LRBA gene: a heterozygous novel frameshift mutation (c.3647_3651delCTAA) as well as an already described mutation (c.7937T>G: I2646S). The patient’s father was found to harbor one heterozygous c.3647_3651delCTAA mutation. The patient’s mother was a healthy heterozygous carrier of the I2646S mutation. This mutation has been reported to cause LRBA deficiency in the homozygous state. The patient’s brother, i.e., the healthy stem cell donor, was heterozygous for I2646S. Moreover, this heterozygous mutation was the only one found in the post-HSCT DNA samples of the patient (Figure 4).

**Table 3:** **List of variants in coding regions detected by using next-generation targeted sequencing on genomic DNA extracted from peripheral blood before the alloHSCT**.

Gene	Ref allele	Alt allele	Allele frequency	Primary effect|function class	SIFT score	PolyPhen2 score
**LRBA**	**GGTTAGTT**	**GGTT**	**0.5**	**FRAME_SHIFT|NM_001199282**		
RIF1	G	A	1.0	NON_SYNONYMOUS_CODING|NM_001177663		
RIF1	G	A	1.0	NON_SYNONYMOUS_CODING|NM_001177663	1.80E−01	B
RIF1	A	T	1.0	NON_SYNONYMOUS_CODING|NM_001177663	4.10E−01	B
RIF1	C	G	1.0	NON_SYNONYMOUS_CODING|NM_001177663	1.00E+00	B
CD86	G	A	1.0	NON_SYNONYMOUS_CODING|NM_001206925	2.90E−01	B
GATA2	C	T	0.5	NON_SYNONYMOUS_CODING|NM_001145661	4.10E−01	B
TFRC	C	T	0.5	NON_SYNONYMOUS_CODING|NM_001128148	4.00E−02	D
TFRC	C	T	0.5	NON_SYNONYMOUS_CODING|NM_001128148	6.30E−01	B
LRBA	G	A	0.5	NON_SYNONYMOUS_CODING|NM_001199282	6.70E−01	B
LRBA	C	T	0.5	NON_SYNONYMOUS_CODING|NM_001199282	1.80E−01	B
**LRBA**	**A**	**C**	**0.5**	**NON_SYNONYMOUS_CODING|NM_001199282**	**0.00E+00**	**P**
TNFSF13	G	A	0.5	NON_SYNONYMOUS_CODING|NM_001198624	5.80E−01	B
TNFSF12-TNFSF	A	G	1.0	NON_SYNONYMOUS_CODING|NM_172089	1.00E+00	B

*Bold font is used to highlight the pathogenic LRBA variants*.

Lipopolysaccharide-responsive beige-like anchor protein expression was analyzed by flow cytometry in PBMCs from fresh samples. The patient’s brother and parents showed normal LRBA expression comparable to the post-HSCT LRBA expression in the patient and two unrelated healthy controls, confirming that the presence of only one heterozygous variant does not affect the expression of the LRBA protein (Figure 4). Pre-alloHSCT vital cells of this patient were not available; therefore, the LRBA expression levels before transplantation could not be assessed. However, the clinical symptoms of the disease were in line with the diagnosis of LRBA deficiency, and the compound heterozygous mutations were identified as deleterious. Possible alternative diagnoses, such as atypical SCID, CD25 deficiency, IPEX, or CTLA-4-deficiency were ruled out based on the results of functional assays and/or genetic workup.

## Discussion

We report the successful treatment of LRBA deficiency-caused severe infantile enteropathy by alloHSCT using 10/10 HLA-matched bone marrow cells of a clinically healthy LRBA-heterozygous sibling. During a long-term follow-up, we observed a complete remission of all LRBA deficiency-related symptoms in our patient without transplantation-related complications.

Heritable monogenic PIDs with a predominant autoimmune phenotype have overlapping symptoms that lie within the clinical spectrum of IBD. The current classification distinguishes IBD subtypes by age of onset: neonatal IBD (develops before the first 28 days of life), infantile IBD (before 2 years of age), very early-onset IBD (VEOIBD, before 6 years of age), and early-onset IBD (EOIBD, before 10 years of age) (1). There is evidence for a high prevalence of monogenic PIDs among patients with infantile IBD, EOIBD, or VEOIBD, which is in contrast to the multifactorial pathogenesis of IBD in adults (1, 23). Whereas some patients suffer from a combination of the disease-defining symptoms, others might initially only present therapy-refractory enteropathy, delaying diagnosis and application of disease-specific treatment. Early diagnosis of the underlying disease is important in order to provide optimal treatment, and it may be a challenge in such patients. There have been about 50 reported cases of patients diagnosed with LRBA deficiency syndrome; they present a broad spectrum of clinical manifestations, ranging from severe multisystemic immunodysregulative disorder (12, 13, 16, 18) to isolated endocrine disease lacking a clear genotype–phenotype correlation (19). While some of these patients died early because of enteropathy and complications of immunosuppressive treatment, others remained stable with or without systemic immunosuppression.

Maintaining a distinction between immunity and autoimmunity requires a complex interaction of activating and inhibiting factors. CTLA-4, mainly localized in intracellular vesicles of Tregs, is a key regulator molecule in this cascade. By acting as an early negative checkpoint, CTLA-4 exerts a major influence on maintaining self-tolerance (24). There is evidence that LRBA controls CTLA-4 expression by preventing its recycling in lysosomes, thereby supporting its trans-endocytotic function and, as a result, the suppressive capacity of Tregs (15). Some LRBA-deficient patients with detectable levels of residual LRBA were reported to have increased CTLA-4 levels, suggesting a quantitative correlation between CTLA-4 deficiency and residual LRBA protein levels in LRBA-deficient patients (15). However, other LRBA-deficient patients display Treg counts within the normal range (13, 19). Moreover, there have been reports of genetically and functionally confirmed CTLA-4-deficient patients with reduced Treg function who remained asymptomatic in the second decade of life (7). There is also evidence for marked Treg dysfunction and skewing in favor of memory T-cells, intense autoantibody production with marked expansion of T follicular helper cells, and contraction of T follicular regulatory cells in LRBA-deficient patients (16). The extent of a possible quantitative correlation between Treg dysfunction and disease severity in these patients should be evaluated in further studies prior to the initiation of immunosuppressive treatment. A recent study postulated a possible role of the base excision repair enzyme Nei endonuclease VIII-like 3 (NEIL3) deficiency as an additional factor contributing to autoimmunity in the presence of LRBA deficiency (25).

For severely affected patients, a wide range of immunosuppressive treatment has been reported as partially beneficial (3, 4). Abatacept, a CTLA-4 fusion protein, has been shown to have a beneficial effect in some LRBA-deficient patients; however, no long-term data on its efficacy and safety are yet available. Furthermore, chloroquine, a lysosomal inhibitor drug used against malaria, was found to augment CTLA-4 levels *in vitro* in Tregs and activated T-cells of LRBA-deficient patients (15).

However, there is accumulating evidence for early alloHSCT being the only curative therapeutic approach for patients suffering from monogenic PIDs causing severe enteropathy, such as IL10/IL10R deficiency (26), the IPEX syndrome (27, 28), and CTLA-4 deficiency (29).

There are three reported cases of alloHSCT performed in patients from consanguineous families who were retrospectively diagnosed with homozygous LRBA mutations; in all cases, non-myeloablative conditioning regimens including fludarabin in combination with treosulfan, busulfan, or melphalan and serotherapy were used (20–22). One patient experienced a complete resolution of the disease symptoms following alloHSCT, while the second patient displayed various complications, adenoviremia and HHV-6 infection with pancytopenia, suspected graft failure, and alloimmune liver disease. Stable engraftment in this patient was ultimately achieved after the application of a stem cell boost and growth factor on day +60. Immune thrombocytopenia (ITP) and vitiligo relapsed in this patient four years post-HSCT after the termination of the immunosuppressive treatment, necessitating intermittent ITP-treatment with romiplostim (21). Post-HSCT ITP was also observed in another patient with the same underlying LRBA mutation as the second patient (c.7162delA). Both patients received stem cells from an HLA-identical heterozygous family donor and achieved full donor chimerism without obvious signs of acute or chronic GVHD. The authors concluded that grafts from donors harboring heterozygous LRBA mutations might induce subclinical autoimmunity; thus, they may not represent the best donor choice. Interestingly, a full remission of thrombocytopenia was observed in both patients, following romiplostim treatment in one patient, and spontaneously in the other patient. During the long-term follow-up period, no further LRBA-associated disease manifestations were observed in either patient. Our results are in keeping with these previous studies and confirm that enteropathy and autoimmunity in LRBA deficiency can be corrected completely by alloHSCT. However, it remains unclear whether LRBA-heterozygosity in clinically healthy stem cell donors has any effect on the status of autoimmunity as a residual disease in transplanted patients. In our case, post-HSCT-heterozygosity did not induce any disease-related symptoms.

Young age lowers the risk of transplantation-related mortality (TRM) because it is associated with lower comorbidity and less frequent end-organ damage caused by the underlying disease. Careful selection of the pre-transplantation conditioning regimen can significantly reduce early TRM in certain PIDs and increase the probability of stable engraftment in others (7).

Due to the lacking genotype–phenotype correlation in LRBA-deficient patients, further data are necessary to establish predictive and prognostic biomarkers that would allow the reliable identification of candidates for early alloHSCT. Further studies on transplantation in LRBA deficiency are necessary to evaluate the optimal conditioning regimen and determine the necessary level of chimerism for disease remission.

## Author Contributions

All authors contributed to the conception and interpretation of the data. SB, PB, AJ, JS, TK, CR, MA, BB, SK, and K-MK provided clinical data. SB wrote the manuscript. LG-D and BG provided data on functional LRBA testing and genetics.

## Conflict of Interest Statement

The authors declare that the research was conducted in the absence of any commercial or financial relationships that could be construed as a potential conflict of interest.
